# A Study of Prognostic Factors in Young Patients With Non-HPV Oral Cancer in Central Europe

**DOI:** 10.3389/pore.2021.1609991

**Published:** 2021-12-22

**Authors:** Katalin Csurgay, Attila Zalatnai, Márta Benczik, Benedek Krisztián Csomó, Ferenc Horváth, Ádám Lőrincz, György Komlós, Zsolt Németh

**Affiliations:** ^1^ Department of Oro-Maxillofacial Surgery and Stomatology, Semmelweis University, Budapest, Hungary; ^2^ 1st Department of Pathology and Experimental Cancer Research, Semmelweis University, Budapest, Hungary; ^3^ Synlab Genoid Molecular Diagnostic Laboratory, Budapest, Hungary; ^4^ Department of Conservative Dentistry, Semmelweis University, Budapest, Hungary; ^5^ Department of Public Health, Semmelweis University, Budapest, Hungary; ^6^ Department of Oral Surgery, King’s College, London, United Kingdom

**Keywords:** immunohistochemistry, young adult, risk factors, oral cancer, p16

## Abstract

The etiological factors of squamous cell carcinomas of the head and neck have been well known for a long time. It is also well known that the incidence of oral cancer diagnosed in younger patients is on the rise. Due to the young age of these patients, the increase in the number of these cases and the fact that many of them neither smoke nor drink alcohol it has been suggested that other factors might be at play in the carcinogenesis of oral cancer. Thus, along the classic etiological factors of smoking and alcohol abuse certain molecular marker anomalies and the human papilloma virus (HPV) have emerged as potential factors. The aim of the present study is to verify the potential prognostic factors and to map the differences in biomarker expression between the young and the old patient groups. In the present study the immunohistochemical profile of samples obtained from oral squamous cell carcinomas was studied and compared with various clinico-pathological parameters. In 88 samples the expressions of p16, p53, Ki67, EGFR were studied with a tissue microarray technique under standard reaction conditions as well as the detection and typing of HPV infection with the Full Spectrum HPV DNA method. The biomarker expression profile of young patients with oral squamous cell carcinoma was compared to that of older patients (above 50). A significant difference was found between the immunohistochemical profile of the young and old patient groups in p16, Ki67 expression. The overall survival and progression free survival were influenced by p16 expression in young age.

## Introduction

Based on the WHO Globocan online database the incidence of oral and lip cancer in 2018 is at the 16th place. Looking at the frequency of all tumours in a population this rank is 12th in the population younger than 50 years. In the same time interval in terms of prevalence oral and lip tumours were ranked 14th and they were in the 11th position among patients under 50 years of age [1]. While tumours induced by alcohol and tobacco use typically occur well over 50, oral tumours of various causes occur in ever younger patients [2].

Changes in molecular markers and HPV have been suggested in this young patient group as important factors or as a viral cause [3, 4].

A change in sexual behaviour is considered to be a risk factor of viral infections, but it remains an open question whether or not the steadily growing number of oral SCCs in young adults can be explained with this alone or not.

Numerous studies suggest that HPV is rarely detectable in the oral tumours that are in the focus of the current study, while it plays a crucial role in the pathogenesis of oropharyngeal tumours [5, 6, 7].

Several studies have attempted to prove that young patients with oral cancer had a different genetic profile compared to older patients with the same disease [8, 9]. P16 positivity could be one such difference suggesting a better prognosis.

It still remains a debated issue, however, whether the above mentioned etiological factors explain the increasing incidence of these cancers at a young age and whether age has a prognostic value or not [10, 11]. In a previous study by the same group the conclusion was reached that age is an independent prognostic factor [12]. Younger patients present sooner with an earlier stage tumour. Relapse occurs sooner and faster, tumour specific survival is shorter. These tumours are characterised by more aggressive spread and early relapse. The primary aim of the present study is to perform a wide survey of the possible prognostic factors of oral cancers presenting in a young adult age group, to identify patients at risk. The basic hypothesis is that instead of tobacco smoking and alcohol consumption [13], changes in protein expression profiles will be found as etiological factors in the young group. It is known that p16 associated tumours have a better prognosis. If p16 is at play in the current study cohort then better survival indicators should be found in the present study, too. Several studies have shown that an increased expression of EGFR, and Ki67 and a mutation in p53 indicate a poor prognosis, which might help screen patients at risk [14].

## Materials and Methods

### Patients

Patients presenting to the Department of Oro-Maxillofacial Surgery and Stomatology of the Faculty of Dentistry, Semmelweis University, Budapest between 2013 and 2018 were included in the study based on strict inclusion criteria. The study was partly retrospective and partly prospective. Inclusion criteria were the following. In terms of location only tumours of the tongue, floor of the mouth and buccal mucosa were included that were surgically removable, had T1-4a, N0-3, M0 stage and were squamous cell carcinomas. No factor in the medical history contraindicating surgery, chemotherapy or radiation therapy was allowed. Study and control groups were formed based on age. Patients younger than 50 years (≤50) were placed into the study group and patients older than 50 into the control group. 101 patients were primarily included, 68 men and 33 women. Six patients were 30 years old or younger. During testing two patients were excluded because of uncertainties in interpreting the immunohistochemical test and 11 further patients due to various other reasons. Following exclusions 50 patients remained in the control group (>50) and 38 in the study group (≤50). Male/female ratio was 61/27. The youngest patient was 22, the oldest 85 years of age.

### Applied Methods

Apart from routine clinico-pathological parameters, the protein expressions of p16, p53, Ki67 and EGFR in the two patient groups were studied with immunohistochemistry.

An appropriate region of the tumour was selected from the specimen embedded in paraffin, tissue cylinders were formed out of these, they were then built into a TMA block and the immunohistochemical reactions performed on them.

### Immunohistochemical Studies

Immunohistochemistry was performed on 4 μm thick, formalin fixed, paraffin-embedded (FFPE) sections of tissue. Slides were deparaffinated with xylene and were hydrated in a series of alcohol baths. Endogenous peroxidase activity was blocked by incubation in 3% hydrogen peroxide for 30 min at room temperature. Following this the slides were washed with a TBS buffer (pH 7.4) for 30 min and were then incubated with the primary antibodies against p16, EGFR, Ki67 and p53. p16 (Santa Cruz Biotechnology, Dallas, United States), EGFR (Santa Cruz Biotechnology, Dallas, United States) and Ki67 (Roche Diagnostics, Basel, Switzerland) antibodies were applied in a dilution of 1:100, while p53 (Roche Diagnostics, Basel, Switzerland) was applied in a dilution of 1:200. For the detection of the immunoreaction the Bond Polymer Refine Detection (Cat. No.: DS9800) reagent was used (Leica Biosystems Ltd., Newcastle, United Kingdom). The kit can be used on a Bond automatic device, which is polymer based with a biotin-free detecting system with DAB. The slides were counterstained with haematoxylin, followed by blotting, drying and mounting [15].

### HPV Screening

HPV detection and type identification were performed from FFPE samples with the Full Spectrum HPV DNA Amplification and Detection method, described in details by Jeney et al. [16]. The method is briefly a multiplex PCR reaction followed by a post-PCR solid phase hybridisation step to detect specific PCR products. The amplification system targets a special hypervariable region of the L1 gene, where a highly conserved and a highly variable region is present in the case of all HPV types [16]. The test can identify 48 different types in groups: five low-risk (HPV6, 11, 42, 43, 44), 14 high-risk (HPV16, 18, 31, 33, 35, 39, 45, 51, 52, 56, 58, 59, 66, 68) and 29 uncategorised risk HPV types (HPV2, 3, 7, 10, 13, 26, 27, 28, 29, 30, 34, 40, 53, 54, 57, 61, 67, 70, 72, 73, 74, 81, 82, 83, 84, 85, 89, 90, 91); as well as able to qualitatively analyse 16 various types of HPV independently (14 high-risk HPV types and two low-risk HPV types: HPV6, 11). Sample DNA preparation was done with an automatic paramagnetic silica based DNA preparation method [16] adapted to TECAN EVO 2000 liquid handling robot from samples obtained from the pathological areas following deparaffination. Human cellularity control was used to verify that the sample DNA can be amplified and that a sufficient number of cells is present in the sample.

### Assessment

**FIGURE 1:**
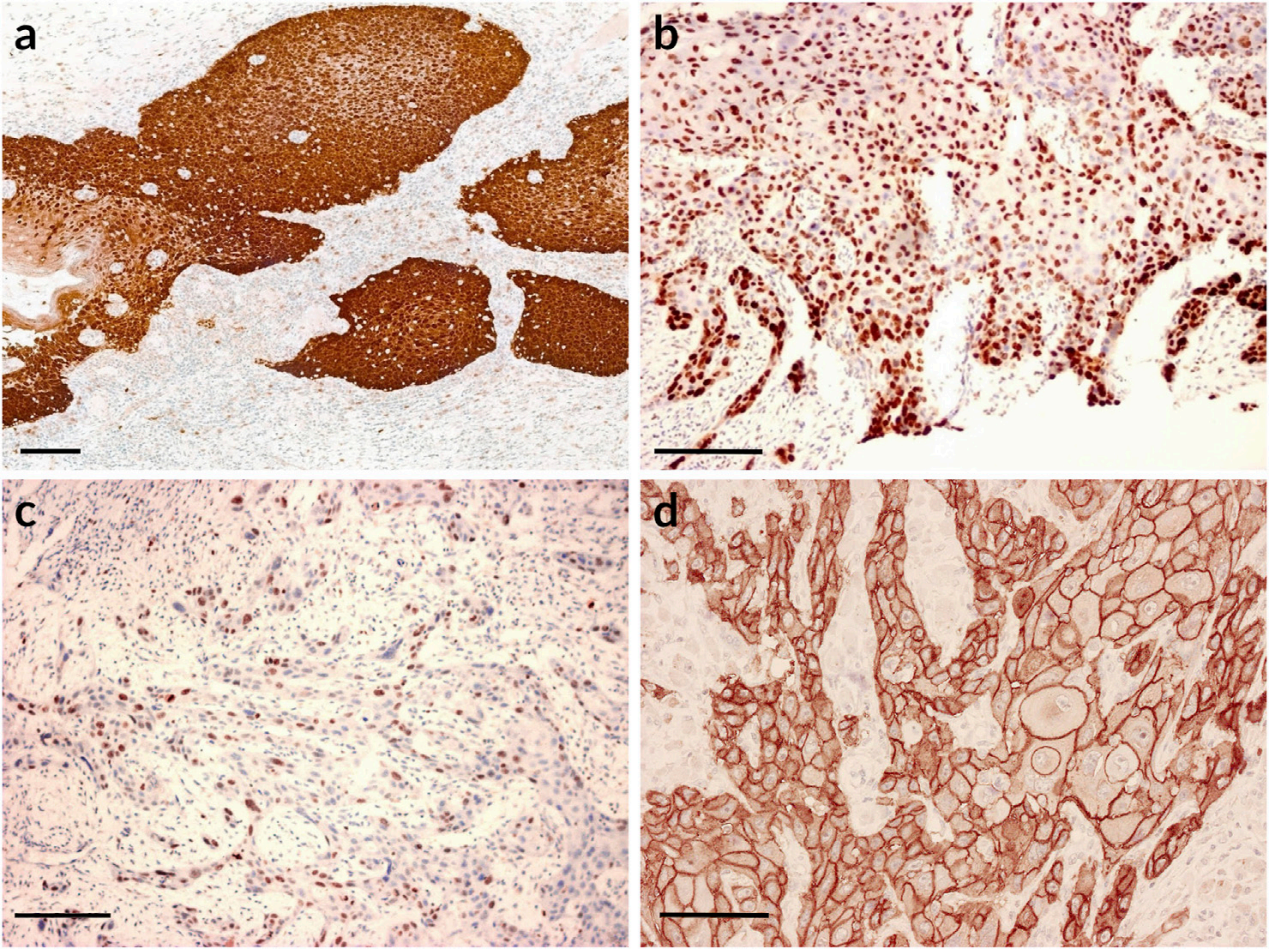
Immunohistochemical expression of p16, p53, Ki67, EGFR. Scale bars: 100 μm. **(A)** p16 positive staining. Immunohistochemistry shows strong p16 staining; **(B)** p53 positive staining. The vast majority of the tumour cells express p53 positivity; **(C)** Ki67 staining. The peripheral cells of the tumorous nests display strong Ki67 nuclear positivity; **(D)** Strong EGFR staining.

Immunohistochemistry was evaluated under a microscope. For p16, Ki67, and p53 the slides were deemed positive or negative and the positivity of the staining was evaluated as a percentage (Figures 1A–C).

For the evaluation of EGFR an H score system was used that assesses the intensity of epithelial staining and percentage ratio (Figure 1D). The value of intensity is ***0***, if there is no staining; ***1***, if slight membrane reaction (+); ***2***, if moderately strong membrane reaction (++); ***3***, if strong membrane positivity (+++) is seen. The percentage of areas of a certain intensity was multiplied by the value of intensity. The product can range from 0 to 300 [17].

All samples were assessed by the same person with the same method.

### Statistical Analyses

Statistical analyses were performed using the IBM SPSS Statistics 27 software. *Fisher’s exact test* was used to study the paired independence of categoric variables. For comparing two groups with a non-normal distribution a *Mann-Whitney U* test was applied. For cross table analyses Pearson’s Chi-square test was used. Regression analysis was done as Cox regression and binomial logistic regression. Linearity of the continuous variables was assessed *via* the *Box-Tidwell* procedure. Estimates were corrected for multiple comparisons with *Bonferroni* corrections. Kaplan Meier (log rank test) and Cox regression were also used for survival analysis.

In univariate regression analysis the variables used were clinico-pathological parameters (gender, age, location, smoking, drinking habits, recurrence, stage, tumour size, lymph node involvement, chemo- and radiotherapy), and immunohistochemical markers (p16, p53, Ki67, EGFR). The variables that were found to be significant in the univariate analysis were included in the multivariate regression model. The only exception were chemo and radiotherapy as the current study did not include the exact doses and drugs used. As regards the IHC markers, threshold values were determined based on the 25th and 50th percentile values.

## Results

### Clinico-Pathological Parameters and IHC Markers

Characteristics of the entire patient population (*n* = 88) can be found in Table 1.

**TABLE 1:** Characteristics of the entire study population.

Parameters		≤50 years n (%)	>50 years n (%)	Total *n* = 88	*p* Values
Gender	Men	27 (71.1%)	34 (68%)	61 (69.3%)	p = 0.758
	Women	11 (28.9%)	16 (32%)	27 (30.7%)	*Pearson χ* ^*2*^
Age (years)	Median	43.5	60		
	Minimum	22	51		
	Maximum	50	85		
Location	Lingual	22 (57.9%)	21 (42%)	43 (48.9%)	p = 0.266
	Floor of the mouth	14 (36.8%)	27 (54%)	41 (46.6%)	*Fisher’s exact*
	Buccal	2 (5.3%)	2 (4%)	4 (4.5%)	
Tobacco	Ever-smokers	27 (71.1%)	38 (79.2%)	65 (75.6%)	p = 0.384
	Never-smokers	11 (28.9%)	10 (20.8%) 2 unknown	21 (24.4%) 2 unknown	*Pearson χ* ^*2*^
Alcohol	Drinkers	12 (31.6%)	18 (38.3%)	30 (35.3%)	p = 0.519
	Non-drinkers*	26 (68.4%)	29 (61.7%) 3 unknown	55 (64.7%) 3 unknown	*Pearson χ* ^*2*^
Recurrence	Yes	20 (52.6%)	20 (40%)	40 (45.5%)	*p* = 0.238
	No	18 (47.4%)	30 (60%)	48 (54.5%)	*Pearson χ* ^*2*^
Stage	I–II	15 (39.5%)	31 (62.0%)	46 (52.3%)	***p* = 0.036**
	III–IV	23 (60.5%)	19 (38%)	42 (47.7%)	*Pearson χ* ^*2*^
T	T1	26 (68.4%)	33 (66%)	59 (67%)	*p* = 0.811
	T2-3-4a	12 (31.6%)	17 (34%)	29 (33%)	*Pearson χ* ^*2*^
N	N0	17 (44.7%)	32 (64%)	49 (55.7%)	*p* = 0.072
	N1–N2	21 (55.3%)	18 (36%)	39 (44.3%)	*Pearson χ* ^*2*^
Chemotherapy	Yes	10 (27%)	12 (24%)	22 (25.3%)	*p* = 0.748
	No	27 (73%)	38 (76%)	65 (74.7%)	*Pearson χ* ^*2*^
		1 unknown		1 unknown	
Radiotherapy	Yes	21 (55.3%)	23 (46%)	44 (50%)	*p* = 0.389
	No	17 (44.7%)	27 (54%)	44 (50%)	*Pearson χ* ^*2*^
Outcome	Dead	19 (50%)	26 (52%)	45 (51.1%)	*p* = 0.853
					*Pearson χ* ^*2*^

*This group includes patients who drink no alcohol and those whose alcohol consumption does not exceed 250 ml 11–13% wine or 4–6% beer occasionally. The significant values indicated with bold letters.

As seen in Table 1 the significant differences between the two patient groups were found in tumour stage, and in protein expression of p16 and Ki67.

A large number of studies conclude that in the younger generation smoking and alcohol consumption are less important etiological factors [18]. In the current study however, no association was found between the age and smoking and alcohol consumption of the study and the control groups. There is no significant difference between the smoking and alcohol consumption of the group younger than 50 and the group older than 50 (Table 1). It seems based on this that the younger generation has similar unhealthy habits as the older generation.

A binomial logistic regression was performed to ascertain the characteristics of the two groups as regards p16, p53, Ki67 and EGFR expression, smoking, alcohol, tumour recurrence and gender. The logistic regression model was statistically significant, χ^2^(9) = 52.653, *p* < 0.001. The model explained 61.8% (Nagelkerke R2) of the variance. Of the nine variables only two were statistically significant: p16 and Ki67. Increasing p16 was associated with a younger age group and increasing Ki67 was associated with an older age group. The risk of recurrence was found to be higher in the group of younger patients than in that of older patients. This was found to be a strong trend.

### Immunohistochemical Markers

**FIGURE 2:**
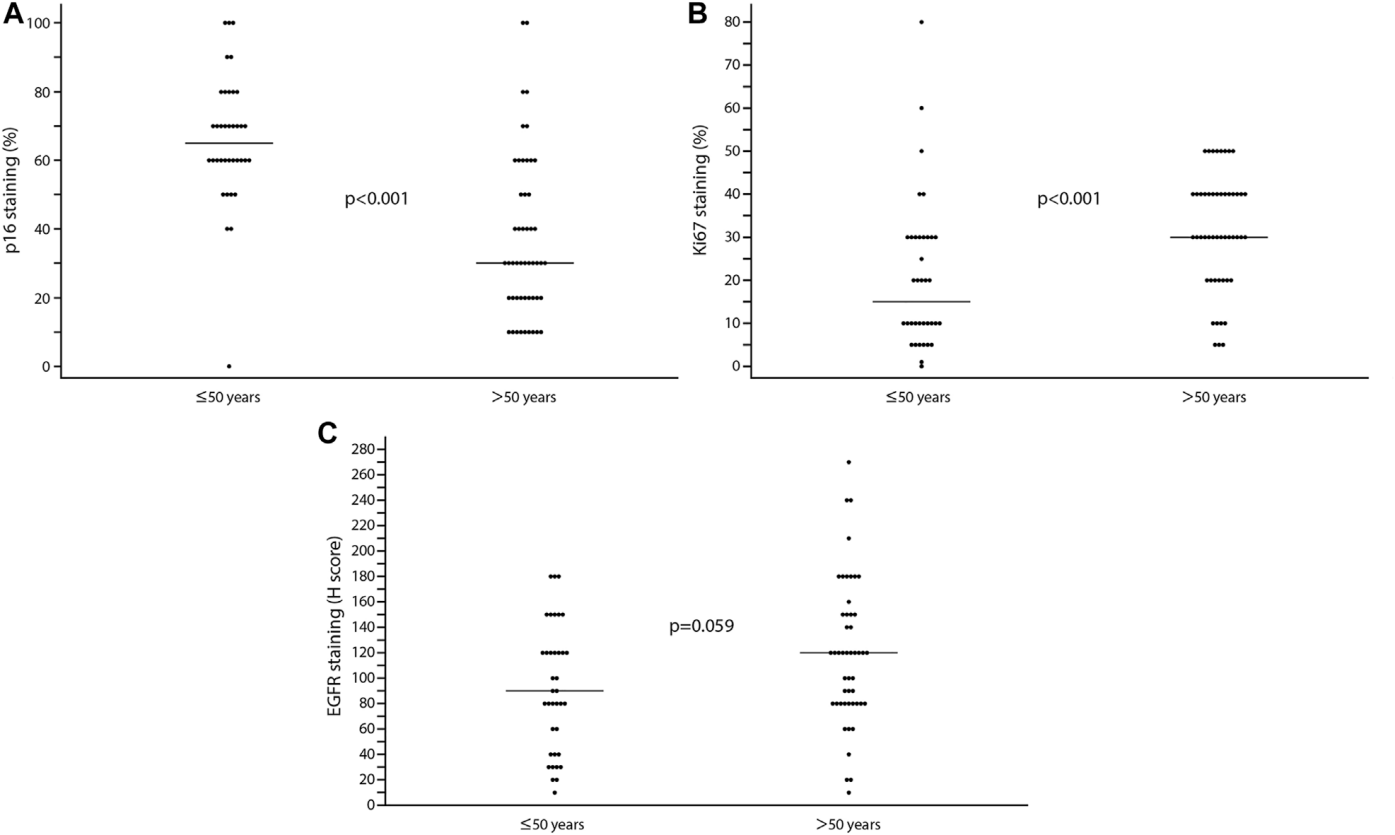
**(A)** The presence of p16 in the study and control groups. The presence of p16 reaches higher levels in young adults. **(B)** Ki67 expression in the two groups. The presence of Ki67 shows a higher value in patients aged over 50. **(C)** A higher value of EGFR can be observed in the control group compared to the study group. The difference has strong tendency for EGFR. The thin horizontal line is the median.

The expression of the tumour suppressor protein p16 was higher in the younger patient group (*p* < 0.001, Mann-Whitney U) (Figure 2A).

Ki67 and EGFR show higher values in the group older than 50 and the differences are significant for Ki67 and have strong tendency for EGFR (Ki67: *p* < 0.001, EGFR: *p* = 0.059, Mann-Whitney) (Figures 2B,C).

When looking at the association between p53 and age groups, the difference was not significant (*p* = 0.364, Mann-Whitney).

### Results of the Full Spectrum HPV DNA Analysis

HPV DNA was expressed in one case only with the Full Spectrum HPV DNA PCR. There were negative results among young patients in the study group. In comparison there was only one positive result with the identification of the high-risk HPV 56 type in the group older than 50. The single HPV positive case was excluded from further statistical analyses.

### Factors Influencing Survival—Survival Analyses

#### Progression Free Survival

A log rank test was run to determine if there were differences in the time to recurrence for the study and control groups. The time to recurrence distributions for the two groups were not statistically significantly different, [Kaplan-Meier, χ^2^(1) = 1.146, *p* = 0.284].

A significant difference was found in the cases of chemotherapy and radiotherapy during the univariate Cox regression analysis (Table 2). The risk of recurrence was found to be higher in patients who received chemo- and radiotherapy. This result is probably due to the fact that patients with a worse prognosis were put on this regime.

**TABLE 2:** Progression free survival analysis with univariate Cox regression.

	Progression free survival (PFS)		
	univar.cox reg.		
	Hazard ratio	95% CI	*p* Values
Gender	0.713	0.376–1.354	0.301
ref: female			
Age	1.401	0.753–2.605	0.287
ref: over 50			
Location			
ref: lingual			
Buccal	2.026	0.607–6.762	0.251
Floor of the mouth	0.580	0.298–1.129	0.109
Smoking	0.684	0.347–1.345	0.271
ref: never-smokers			
Alcohol	1.141	0.593–2.195	0.694
ref: non-drinkers			
Stage	1.466	0.787–2.729	0.228
ref: stage I, II			
T	1.206	0.630–2.311	0.572
ref: T1			
N	1.531	0.823–2.848	0.179
ref: N0			
Chemotherapy	4.688	2.472–8.891	**<0.001**
ref: no			
Radiotherapy	5.294	2.509–11.170	**<0.001**
p16			
ref: <60%			
p16	0.722	0.385–1.351	0.308
(60%<)			
p53			
ref: <50%			
p53	0.823	0.441–1.534	0.539
(50%<)			
Ki67			
ref: <30%			
Ki67	0.707	0.380–1.315	0.274
(30%<)			
EGFR			
ref:<90 H score			
EGFR	2.307	1.127–4.725	**0.022**
(H score 90-300)			

The significant values indicated with bold letters.

Out of protein expressions EGFR showed a significant correlation with disease progression. It was found that the risk of relapse is higher if EGFR has a value above 90 H score (Table 2).

The multivariate analysis yielded the result that the risk of recurrence was higher in the group younger than 50, than in that of patients older than 50 (Table 3).

**TABLE 3:** Progression free survival analysis with multivariate Cox regression.

	Hazard ratio	95% CI	*p* Values
Age	2.772	1.068–7.194	**0.036**
ref: over 50			
Smoking	0.672	0.316–1.432	0.303
ref: never-smokers			
Alcohol	1.154	0.547–2.321	0.687
ref: non-drinkers			
Ki67	0.603	0.283–1.287	0.191
ref:<30%			
p53	0.732	0.375–1.428	0.360
ref:<50%			
p16	0.271	0.108–0.681	**0.005**
ref: <60%			
EGFR	3.141	1.465–6.734	**0.003**
ref:<90 H score			
T	1.431	0.707–2.894	0.319
ref: T1			
N	1.792	0.911–3.525	0.091
ref: N0			

The significant values indicated with bold letters.

Based on the multivariate Cox regression analysis a significant difference was found in p16 and EGFR protein expressions. The risk of recurrence was found to be lower if p16 staining was above 60% than if it was under this value, this result is significant. It was also found that the risk of recurrence was significantly higher if EGFR staining was above 90 H score.

**FIGURE 3:**
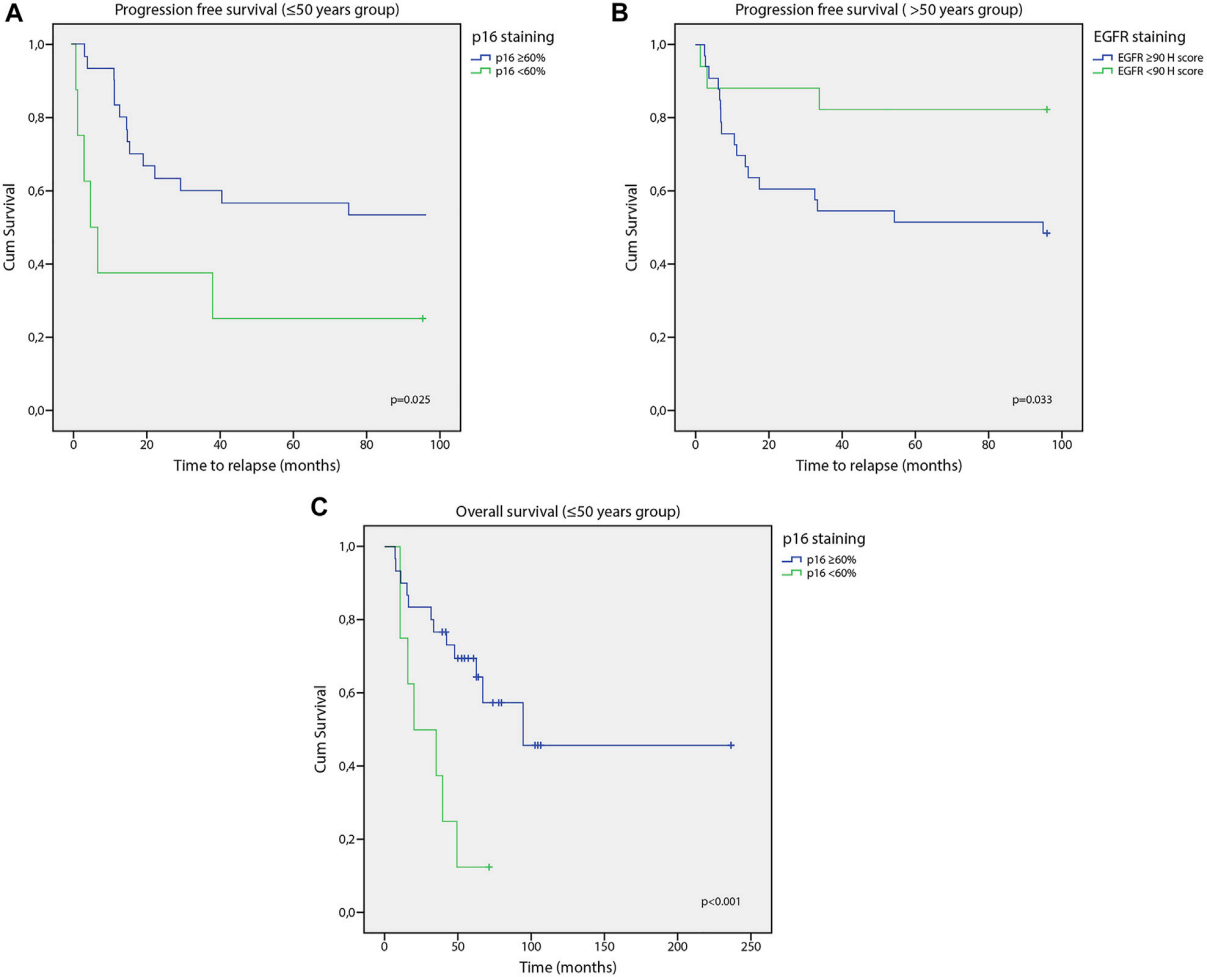
**(A)** Correlation of p16 staining and progression free survival. P16 expression over 60% was better progression free survival in the younger than 50 group. **(B)** Correlation of EGFR staining and progression free survival. PFS was significantly worse with an EGFR over 90 H score. **(C)** Correlation of p16 staining and overall survival. P16 value above 60% means better survival in the under 50 group.

Analysing the subgroups, it was found that tumours with N1-N2 lymph node metastases predict worse progression free survival than an N0 situation in the younger than 50 group [Kaplan-Meier, χ^2^(1) = 5.427, *p* = 0.020)] In a p16 expression over 60% in the younger than 50 group progression free survival was significantly better [Kaplan-Meier, χ^2^(1) = 5.057, *p* = 0.025] (Figure 3A).

In the older than 50 group with an EGFR over 90 H score progression free survival was significantly worse [Kaplan-Meier, χ^2^(1) = 4.532, *p* = 0.033] (Figure 3B), and was worse in drinkers, too [Kaplan-Meier, χ^2^(1) = 3.869, *p* = 0.049].

Progression free survival was significantly worse in both groups if the patients received chemo- or radiotherapy [chemotherapy <50 Kaplan-Meier, χ^2^(1) = 13.222, *p* = 0.000; ≥50 Kaplan-Meier, χ^2^(1) = 13.935, *p* = 0.000; radiotherapy <50 Kaplan-Meier, χ^2^(1) = 7.749, *p* = 0.005; ≥50 Kaplan-Meier, χ^2^(1) = 16.089, *p* = 0.000].

No significant correlation was found between sex, smoking, tumour size, stage, p53 or Ki67 and progression free survival in the subgroup analysis.

#### Overall Survival

A log rank test was run to determine if there were differences in the survival distribution for the study and control groups. The survival distributions for the two groups were not statistically significantly different, [Kaplan-Meier, χ^2^(1) = 0.309, *p* = 0.578].

Survival of patients drinking alcohol regularly was found to be worse than those who only consume alcohol socially or those who do not drink alcohol at all (Table 4).

**TABLE 4:** Overall survival analysis with univariate Cox regression.

	Overall survival (OS)		
	univar.cox reg.		
	Hazard ratio	95% CI	*p* Values
Gender	1.348	0.691–2.627	0.381
ref: female			
Age	0.816	0.448–1.483	0.504
ref: over 50			
Location			
ref: lingual			
Buccal	1.401	0.326–6.027	0.650
Floor of the mouth	1.145	0.628–2.087	0.659
Smoking	1.277	0.611–2.670	0.516
ref: never-smokers			
Alcohol	1.974	1.070–3.640	**0.029**
ref: non-drinkers			
Recurrence	1.773	0.983–3.199	0.057
Stage	2.305	1.257–4.227	**0.007**
ref: stage I, II			
T	1.760	0.975–3.178	0.061
ref: T1			
N	2.238	1.235–4.054	**0.008**
ref: N0			
Chemotherapy	2.792	1.507–5.170	**<0.001**
ref: no			
Radiotherapy	1.461	0.808–2.642	0.210
p16			
ref: <60%			
p16	0.793	0.436–1.442	0.447
(60%<)			
p53			
ref: <50%			
p53	0.763	0.420–1.385	0.374
(50%<)			
Ki67			
ref: <30%			
Ki67	1.017	0.562–1.838	0.957
(30%<)			
EGFR			
ref:<90 H score			
EGFR	0.940	0.518–1.708	0.840
(H score 90–300)			

The significant values indicated with bold letters.

The link between stage and survival is also significant. Survival in stages III–IV is worse than in stage I–II.

The correlation between overall survival and lymph node involvement (N) as established with Cox regression was significant. Tumours with N1 and N2 lymph node metastases predict worse survival than an N0 situation.

The survival of patients receiving chemotherapy was significantly worse, the correlation is significant. This can mean that chemotherapy is administered to patients with worse prognosis, thus the survival rate is lower. On the other hand the toxicity of chemotherapy may also cause worse survival rates. The analysis of this issue is not given in the present study, as the study was not designed to investigate this issue. Further investigation would be necessary to clarify the issue (Table 4).

With multivariate analysis lymph node involvement also showed a significant correlation with survival (Table 5). The survival of N1–N2 tumours was worse than in N0 tumours.

**TABLE 5:** Overall survival analysis with multivariate Cox regression.

	Hazard ratio	95% CI	*p* Values
Age	0.899	0.353–2.291	0.824
ref: over 50			
Smoking	1.212	0.529–2.775	0.650
ref: never-smokers			
Alcohol	1.867	0.932–3.743	0.078
ref: non-drinkers			
Ki67	0.824	0.373–1.819	0.632
ref:<30%			
p53	0.684	0.357–1.311	0.253
ref:<50%			
p16	0.715	0.303–1.684	0.442
ref: <60%			
EGFR	0.929	0.489–1.764	0.822
ref:<90 H score			
T	1.626	0.852–3.101	0.140
ref: T1			
N	2.363	1.219–4.582	**0.011**
ref: N0			

The significant values indicated with bold letters.

Analysing subgroups with a Kaplan Meier log rank test it was found that in the under 50 group survival tends to be worse in case of disease recurrence. This has a strong tendency. [Kaplan-Meier, χ^2^(1) = 3.510, *p* = 0.061]. Survival is significantly better in stage I, II than in stage III, IV in both groups [<50 Kaplan-Meier, χ^2^(1) = 4.144, *p* = 0.042; ≥50 Kaplan-Meier, χ^2^(1) = 5.835, *p* = 0.016]. As in PFS, a p16 value above 60% means better survival in the under 50 group [Kaplan-Meier, χ^2^(1) = 8.128, *p* = 0.004] (Figure 3C). Survival is significantly worse in both the younger and older patient groups in case of chemotherapy [<50 Kaplan-Meier, χ^2^(1) = 5.758, *p* = 0.016; ≥50 Kaplan-Meier, χ_2_(1) = 6.399, *p* = 0.011] and in case of N1-N2 tumours [<50 Kaplan-Meier, χ^2^(1) = 4.516, *p* = 0.034; ≥50 Kaplan-Meier, χ^2^(1) = 4.402, *p* = 0.036]. No significant correlation was found between sex, tumour size, drinking, smoking, radiotherapy, p53, EGFR or Ki67 and survival in the subgroup analysis.

## Discussion

It has already been proven that regular smoking and alcohol abuse are major factors in the pathogenesis of most oral cancers. In the literature it is often stated that these classic etiological factors do not have a huge impact on tumour evolution in young patients. Certainly, at least 10–20 years of regular smoking and heavy drinking are required for the development of oral cancer. There is no chance for such a prolonged exposure time among younger patients. For instance, in a recent study a 22-years-old patient was diagnosed with oral cancer [19, 20]. In comparing the younger and older oral cancer patient group there was no significant difference in tobacco and alcohol consumption. It can also be stated that the number of smokers is high in both the older and younger oral cancer patient groups. Contrary to the results of the international literature, the occurrence rate of harmful lifestyle habits is similar in younger and older patient groups in the Hungarian oral cancer patient population. The patients were predominantly male, although the youngest patient was female (22 years old). When studying tumour site and age there does not seem to be a preference for certain locations at certain ages suggesting sexual activity or customs. A significant difference was found between the younger (under 50) and older (over 50) patient groups when studying tumour stage, p16 and Ki67 protein expression. It was found that p16 is expressed more frequently in the younger than 50 group. Ki67 had a significantly higher value in the over 50 group. EGFR expression values were also higher in the older group, but this is only a nearly significant trend.

Tamás et al. described correlations between biomarker expressions (Ki67 and EGFR) and tumour location. EGFR expression was the highest in cancers of the oral cavity and the lowest in glottis cancers. These results could not be reproduced in the current study as strictly only oral cancers were included [21, 22].

Adduri et al. found a significant correlation between p53 nuclear stabilisation and a young age. The present study did not uncover such a correlation with p53 expression, which might be due to the different genetics of the populations [23].

Recurrence of the tumour is more frequent in younger oral cancer patients, which suggests that age might be a predisposing factor for OC development [19]. In this manner, there is a need for a more frequent follow up in younger OC patients. Our study could identify age as a risk factor, as in the multivariate analysis of progression free survival it was found that the risk of recurrence was higher in the younger patient group than in the older one. In addition to age p16 and EGFR protein expression have been found to effect progression. Overall survival seems to be influenced by alcohol consumption, lymph node involvement and stage.

It is known that p16 can be a surrogate marker and can suggest the presence of HPV in the tumour, as the inhibition of the retinoblastoma protein expression of the tumour suppressor gene p16 can be induced [24, 25]. Presence of HPV is expected to predict a better prognosis in head and neck cancer [26, 27].

In the present study HPV could only be detected in one case of the older than 50 group out of many that had p16 positivity [28]. This can be explained by the fact that only oral cancer patients were enrolled in our study. In non-oropharyngeal cancers HPV involvement is five times less frequent than in oropharyngeal cancer [3, 29, 30, 31, 32].

It is also known however, p16 positivity itself is not necessarily proof of HPV involvement. For example, triple negative breast cancer is characterised by strong p16 positivity without the involvement of HPV [33].

Correspondingly, the present study supports that HPV cannot be considered to be the main etiological factor in the development of oral cancer in young age.

p16 expression and the prognosis of cancer are different in various tumour types. In malignant melanoma, nasopharyngeal carcinoma, hepatocellular carcinoma, oesophageal and oropharyngeal squamous cell carcinoma, and in osteosarcoma the reduction of p16 expression is a negative prognostic indicator. On the other hand, in prostate tumours, ovarian cancer, and neuroblastoma an increase in the expression is a negative prognostic factor [34, 35, 36, 37, 38, 39, 40, 41]. HPV is also not an etiological factor in these tumours.

Harris et el. reported significantly better survival rate by increased p16 expression, while Miller et al. reported no correlation between p16 expression and the survival rate in a group of patients with tongue cancer in 2019 [8, 42]. The present study supports that p16 plays a prognostic role in the group of patients under 50 years of age, however the current study population was more complex because of the locations of the OC (on the tongue, floor of the mouth and the buccal mucosa). If p16 staining is above 60%, then overall survival is better at a young age. Statistical analysis has shown that the risk of recurrence is lower when p16 staining is above 60%.

An increased expression of EGFR can be found in almost 90% of oral cancers and it is a negative prognostic indicator [43, 44, 45].

A Japanese group of researchers reported that the high expression of EGFR and p-EGFR was correlated with tumour invasion, however high expression was unrelated to tumour stage, lymph node or distant metastasis [46]. In the present study it was found that an increased expression of EGFR was more common (with a strong tendency) in the group older than 50. The risk of recurrence was found to be higher if EGFR staining was above 90 H score, which indicate the negative prognostic value of EGFR. Multivariate analyses also indicate that an EGFR value above 90 H score reduces the chance of progression free survival. Analysis of the subgroups showed that EGFR had a negative effect on progression free survival in the over 50 group. A number of studies have reported that a higher rate of Ki67 positivity was a negative prognostic factor, but in the current study no significant correlation was found either for Ki67 or for p53 [47, 48].

The results of the multivariate analysis demonstrate that a younger age is a risk factor for progression and also that differences in the immunohistochemical profile (p16, EGFR) have a significant impact on progression.

The results of the present study confirmed the supposed link between immunohistochemical profile changes and oral cancer. The role of p16 protein expression seems to be more important in the group of younger patients with oral cancer, as it influences survival. It can be hypothesised that differences in protein expressions are probably behind the growing incidence of oral malignancies in a younger age, rather than a viral aetiology. The hypothesis that the current study group was independent of classic risk factors could not be confirmed. The results suggest that the study and the control group have the similar lifestyle risk factors. At the same time it can be concluded that alcohol consumption clearly influences overall survival in oral cancer patients. Based on the multivariate analysis it is also clear that age, p16 and EGFR expression play a decisive role in progression free survival. Changes in molecular markers are also important as the study of p16 expression yielded significant differences in the group of OC patients under the age of 50. Survival seems to be better if p16 staining is over 60% in a young age even without the presence of HPV. Ki67 and EGFR showed a tendency of higher values in the group of OC patients above the age 50. EGFR staining over 90 H score is a negative prognostic factor as in these cases the risk of recurrence is higher. The synergistic effects of genetic changes and harmful lifestyle habits would merit further studies, but it can be said that in the case of young patients a better prognosis can be expected if p16 positivity is over 60% in non-HPV tumours. The importance of the current study is that a close correlation between genetic changes and classic etiological factors was studied in homogeneous patient groups.

Study limitations can be summarised as low sample size due to the rarity of these tumours in a young age and to convenient sampling as patients from a university department were included in the study. In addition to immunohistochemistry further RNA gene expressions studies could increase the validity of the results.

## Data Availability

The raw data supporting the conclusions of this article will be made available by the authors, without undue reservation.

## Abbreviations

DAB, Diamino-benzidine; EGFR, Epidermal growth factor receptor; HPV, Human papilloma virus; ISH, *In situ* hybridization; Ki67, Nuclear antigen; p16, cyclin-dependent kinase inhibitor; p53, Nuclear protein; PCR, Polymerase chain reaction; PFS, Progression free survival; Rb, Retinoblastoma protein; TBS, Tris buffered saline; TMA, Tissue microarray; SCBT, Santa Cruz Biotechnology.
